# Longitudinal changes in bleb morphology following the PAUL Glaucoma Implant visualized using ultrasonography

**DOI:** 10.1111/aos.70180

**Published:** 2026-07-22

**Authors:** Constance Liegl, Benjamin Aretz, Leonie Bourauel, Pascal Schipper, Carolin Deubel, Lilli Joussen, Michael Petrak, Frank G. Holz, Raffael Liegl, Karl Mercieca

**Affiliations:** ^1^ Department of Ophthalmology University of Bonn Bonn Germany; ^2^ Department of Ophthalmology, Klinikum Dortmund University Witten/Herdecke Dortmund Germany; ^3^ Institute for Medical Biometry, Informatics and Epidemiology University of Bonn Bonn Germany

**Keywords:** glaucoma drainage device, glaucoma surgery, PAUL Glaucoma Implant, sonography

## Abstract

**Purpose:**

To evaluate bleb morphology longitudinally using sonography following glaucoma drainage device (GDD) surgery.

**Methods:**

Prospective observational cohort study conducted from January 2022 to March 2024, with follow‐up up to 12 months postoperatively. Bleb morphology, including superior and inferior bleb height, transverse and longitudinal dimensions, and total bleb height, was assessed using 15‐MHz ultrasound (Quantel Medical, Compact Touch).

**Results:**

Forty‐eight eyes undergoing PGI implantation were prospectively enrolled. Preoperative mean IOP was 23.6 mmHg (range, 13–42) on 3.2 medications. Mean total bleb height increased from 2.33 mm (range, 0.64–6.6) at day 14 to 4.14 mm (1.21–7.66) at month 3 and remained stable through month 12 (4.21 mm; 2.13–9.4). Double‐layered blebs predominated by month 1 and persisted thereafter. Mean IOP decreased to 12.7 mmHg at day 14 and remained between 13 and 15 mmHg through 12 months, while medication use declined to 0.9 drops. Mixed‐model analysis showed that absolute bleb height was significantly associated with higher IOP (*β* = 0.86, *p* = 0.043), independent of time. Accelerated bleb enlargement within the first 3 months correlated with higher IOP (*β* = 3.57, *p* = 0.003), whereas later changes showed no consistent associations.

**Conclusions:**

Ultrasound imaging revealed progressive bleb enlargement after PGI implantation, stabilizing by 3–6 months. In the subgroup of patients who completed the 12‐month follow‐up, IOP reduction remained stable throughout the first postoperative year. Absolute bleb height showed a consistent association with IOP across time, whereas early growth rate was only predictive during the early postoperative period. These findings provide the first longitudinal sonographic evidence of bleb remodelling after GDD surgery and may guide postoperative assessment and intervention timing.

## 1 INTRODUCTION

Glaucoma drainage devices (GDDs) are commonly used in eyes with secondary glaucoma—such as uveitic or neovascular types—and in cases of primary open‐angle glaucoma (POAG), particularly when previous surgeries have failed or conjunctival scarring limits other surgical options (Aref et al., 2017; Lee et al., 2020; Pereira et al., 2021). The PAUL® Glaucoma Implant (PGI) resembles the Baerveldt tube in size and valveless design but features a smaller tube diameter and a unique plate configuration and material. Published studies with up to three‐year data report effective IOP reduction, reduced medication use and a low rate of postoperative hypotony.

A critical component of GDD function is the formation of a filtering bleb around the implant plate in the post‐equatorial region. Due to its posterior location, conventional slit‐lamp examinations are inadequate for assessing these blebs, necessitating alternative imaging techniques. Although magnetic resonance imaging is currently the most established method for visualizing GDD blebs, its high cost and limited availability restrict its routine clinical use (Vallabh et al., 2021). Ultrasonography offers a more accessible and practical alternative; however, existing studies on its application in evaluating GDD blebs are limited and largely cross‐sectional (Anderson et al., 2017; Correia Barão et al., 2023; Duan et al., 1997; Lloyd et al., 1993; Sano et al., 2015). To date, no study has systematically examined the longitudinal development of GDD blebs over time—a key gap, as such information could be important for guiding and tailoring postoperative management. Understanding how blebs evolve in the first 6–12 months after surgery may help optimize therapy and improve long‐term outcomes. This study aims to address this gap by longitudinally analysing bleb morphology using ultrasonographic imaging and correlating these findings with intraocular pressure (IOP), ultimately contributing to a standardized framework for postoperative evaluation.

## 2 MATERIALS AND METHODS

Patients who underwent PGI surgery at the Glaucoma Service of the University Eye Hospital, Bonn, Germany, between January 2024 and January 2025 were included in this study. At each follow‐up visit—scheduled at 10–14 days, 1 month, 3 months, 6 months and 12 months postoperatively—patients received a standardized 15 MHz B‐scan ultrasound examination (Quantel Medical, Compact Touch, France). The filtering bleb was assessed in both transverse (T) and longitudinal (L) planes, with measurements taken for its extension and height. Bleb configuration (presence of one or two compartments) and axial length (AL) were also recorded. Comprehensive ophthalmic examinations were conducted at each visit, including best‐corrected visual acuity (BCVA) using the Snellen chart (converted to logMAR for analysis), intraocular pressure (IOP) via Goldmann applanation tonometry, slit‐lamp biomicroscopy and fundus examination. Patient records were reviewed to extract preoperative data such as gender, age, glaucoma type, BCVA, IOP and anterior segment findings. Follow‐up data included BCVA, IOP, complications, postoperative glaucoma medications and bleb characteristics.

Inclusion criteria were clear ocular media allowing adequate visualization during sonography, successful implantation of a PGI, and availability of longitudinal sonographic follow‐up data for at least 6 months. Eligible patients were required to be over 18 years of age. Exclusion criteria included incomplete follow‐up data and the presence of intraocular silicone oil, as this precludes reliable sonographic imaging.

The primary outcome of the study was to describe the longitudinal development of the filtering bleb at each postoperative time point. Secondary outcomes included IOP, BCVA, number of IOP‐lowering agents, AL and correlations between bleb characteristics and IOP. Ethics approval was granted from the local Ethics Committee at the University Hospital in Bonn (approval number 458/21 and 2024‐17‐BO). The study protocol conformed to the ethical guidelines of the 2000 Declaration of Helsinki as reflected in a priori approval by the institution's Human Research Committee.

### 2.1 Statistical analysis

Statistical analysis was performed with SPSS Statistics version 27.0.0 (IBM Corporation, New York) and R software environment (version 4.4.3; R Foundation, Vienna, Austria). Continuous variables are presented by the mean, range and standard deviation (SD); categorical variables are presented by the total numbers and percentages.

To investigate the association between postoperative bleb height development and IOP, we estimated two types of linear mixed‐effects regression models with random intercepts at the patient level to account for repeated measurements within patients.

First, level models were specified, in which the absolute total bleb height at each postoperative time point (14 days [± 3 days, thereafter referred to as 14 days], 1, 3, 6 and 12 months) was included as a predictor of IOP. Fixed effects for age, sex, visual acuity (logMAR) and the number of IOP‐lowering eye drops were added as covariates. An interaction term between time point and bleb height was included to assess potential time‐dependent modifications of the bleb–IOP association.

Second, growth models were estimated to capture postoperative growth dynamics. For that purpose, individual growth rates of total bleb height were computed relative to baseline (14 days postoperatively) for each patient. The growth rate was thus defined as the change in total bleb height from baseline (14 days postoperatively) to each respective follow‐up time point, divided by the elapsed time in months (Δ height/Δ time). A higher growth rate, therefore, reflects a faster postoperative increase in bleb height within the corresponding interval, indicating more pronounced structural changes during that period. Separate growth rates were calculated for the intervals 14 days → 1 month, 14 days → 3 months, 14 days → 6 months and 14 days → 12 months. These interval‐specific growth rates were then included as predictors of IOP, together with patients' baseline total bleb height and the same covariates as in the level models. An interaction term between interval and growth rate was incorporated to test whether the impact of growth differed across follow‐up periods.

All models were estimated using restricted maximum likelihood (REML) as implemented in the lme4 package in R. Model assumptions were checked by visual inspection of residual plots.

### 2.2 Surgical technique

All procedures were carried out by a single experienced glaucoma surgeon (KM). In all cases, the PGI was positioned in the superotemporal quadrant.

Surgical exposure was obtained through a fornix‐based conjunctival and Tenon's peritomy. A sponge soaked in mitomycin‐C (0.5 mg/mL) was applied to the implantation area for approximately 2 min to modulate postoperative fibrosis. The implant plate was then placed beneath the rectus muscles and fixed about 10 mm posterior to the limbus with 9‐0 nylon sutures. To prevent early postoperative hypotony, a 6‐0 polypropylene intraluminal stent was inserted into the tube to temporarily limit aqueous flow.

A 26‐gauge needle was used to enter the anterior chamber, through which the tube was inserted via a scleral tunnel, trimmed to the desired length and secured with a box‐shaped 9‐0 nylon suture. The exposed tube was then covered with a double‐layered pericardial patch graft, which was fixed in place using fibrin glue. The free end of the stent was typically tucked into a subconjunctival pocket in the inferior quadrant for later removal. Finally, the conjunctiva was closed using two 10‐0 nylon slip knots at the corners and two interrupted mattress sutures.

Postoperatively, all patients received topical corticosteroid eye drops six times daily, antibiotic eye drops four times daily, and atropine eye drops once daily for 10 days. After 10 days, atropine and antibiotics were discontinued, and corticosteroids were gradually tapered over a three‐month period according to the clinical response.

## 3 RESULTS

### 3.1 Study population

Forty‐eight eyes of Caucasian patients (18 men, 30 women; mean age 64 years, range 19–89) underwent PGI surgery and postoperative ultrasound examinations at 14 days (± 3 days) post‐surgery as well as 6 months after surgery. Baseline characteristics are summarized in Table 1, including glaucoma subtype, prior ocular surgery, preoperative IOP, BCVA and medication use. Mean IOP was reduced from 23.6 mmHg preoperatively to 12.7 mmHg at day 14 and remained between 13 and 15 mmHg throughout follow‐up (month 1: 13.3 mmHg; month 3: 15.6 mmHg; month 6: 14.7 mmHg; month 12: 14.8 mmHg). The number of IOP‐lowering eye drops fell from a preoperative mean of 3.21 to 0.46 at day 14 and remained ≤ 0.9 up to month 12. BCVA was stable, measuring 0.69 logMAR (range 0–1.9) at month 12 compared with 0.56 logMAR preoperatively (Table 1). Prolene removal was required in five eyes (10.4%), all after the first 3 postoperative months.

**TABLE 1:** Demographics and clinical characteristics of eyes undergoing PGI surgery.

	*n* = 48 eyes
Gender	
Male/female	18 (37.5)/30 (62.5)
Ethnicity	
Caucasian	48 (100)
Age	
Mean (range)	64.33 (19–89)
Glaucoma type	
Primary open‐angle glaucoma	25 (52.1)
Pseudoexfoliative glaucoma	2 (4.2)
Secondary glaucoma	19 (39.6)
Others	2 (4.2)
Previous glaucoma surgery	
Yes	21 (43.8)
Which surgery	
Trabeculectomy	7 (14.6)
XEN	2 (4.2)
Canaloplasty	2 (4.2)
Trabectome	2 (4.2)
CPC	10 (20.8)
Deep sclerectomy	3 (6.3)
I Stent	1 (2.1)
Preserflo	3 (6.3)
Previous vitrectomy	
Yes	18 (37.5)
Number (#) of glaucoma drops	
Mean (range)	3.21 (1–5)
Acetazolamide	
Yes	15 (31.3)
Lens status	
Phakic. Pseudophakic; aphakic	10 (20.8)/36 (75)/2 (4.2)
Maximum IOP in medical history	
Mean (range)	33.42 (16–65)
IOP during the postoperative course	
Preoperative	23.62 (13–42)
Day 14	12.73 (6–24)
Month 1	13.26 (6–23)
Month 3	15.55 (7–26)
Month 6	14.66 (9–31)
Month 12	14.75 (6–35)
BCVA during the postoperative course	
Preoperative	0.56 (0–1.9)
Day 14	0.68 (0.1–2.3)
Month 1	0.63 (0.0–1.9)
Month 3	0.53 (0–2.3)
Month 6	0.51 (0–1.9)
Month 12	0.69 (0–1.9)

Amount of eye drops during the postoperative course	
Preoperative	Eye drops
Day 14	3.21 (1–5)
Month 1	0.46 (0–4)
Month 3	0.53 (0–4)
Month 6	0.65 (0–4)
Month 12	0.74 (0–3)
	0.9 (0–3)

### 3.2 Evolution of bleb morphology

Ultrasound revealed progressive enlargement of the filtering bleb over the first 3 months, followed by a stabilization in size (Table 2).

**TABLE 2:** Morphological bleb development and growth rates.

	Day 14 *n* = 48	Month 1 *n* = 41	Month 3 *n* = 40	Month 6 *n* = 48	Month 12 *n* = 21
Superior bleb height	1.79 (0.5–3.5)	1.98 (0.8–4.1)	2.17 (0.6–4.6)	2.01 (0.7–5.5)	2.08 (1.2–5.3)
Superior bleb (T)	23.81 (17.3–38.3)	22.7 (7.4–29.9)	23.74 (17.7–31.3)	23.89 (17.2–31.3)	23.74 (18.6–25.9)
Superior bleb (L)	17.20 (14.6–20.2)	17.3 (11.5–27.2)	18.00 (11.8–27.3)	17.81 (13.3–20.9)	17.3 (14.1–19.3)
Inferior bleb height	1.44 (0.56–4.02)	2.05 (0.64–5.39)	2.07 (0.8–5.9)	2.14 (0.5–5.8)	2.13 (0.8–5.9)
Inferior bleb (T)	23.82 (16.3–31.2)	23.5 (16.3–28.6)	22.89 (16.5–30.2)	23.83 (18.4–30.2)	23.75 (14.9–19.9)
Inferior bleb (L)	17.24 (12.2–23.1)	17.11 (12.2–23.6)	17.11 (1.6–23.5)	18.14 (13.4–25.9)	17.54 (14.9–19.9)
Total height	2.33 (0.64–6.6)	3.79 (0.65–7.0)	4.14 (1.21–7.66)	4.15 (1.45–11.0)	4.21 (2.13–9.4)

Descriptive statistics of growth rates (mm/month) by interval					
Interval	Mean	SD	Median	Min.	Max.
14 days → 1 month	1.56588486	1.84	1.22	−0.75	6.12
14 days → 3 months	0.54682144	0.61	0.49	−0.52	2.07
14 days → 6 months	0.27433871	0.29	0.31	−0.30	1.11
14 days → 12 months	0.08965408	0.17	0.06	−0.19	0.41

Mean total bleb height increased from 2.33 mm (range 0.64–6.6) at day 14 to 3.79 mm (0.65–7.0) at month 1 and 4.14 mm (1.21–7.66) at month 3, remaining essentially unchanged thereafter (month 6: 4.15 mm; month 12: 4.21 mm).

Both superior and inferior bleb heights followed a similar pattern, reaching mean values of 2.01 mm and 2.14 mm, respectively, by month 6 and remaining stable to month 12.

Bleb configuration evolved in parallel. At day 14, approximately 40% of eyes exhibited a single‐layered bleb, while the remainder had already shown a double‐layered configuration. By month 1, the vast majority of eyes displayed double‐layered blebs, a pattern that persisted through to month 12.

The horizontal dimensions showed little change: transversal extension (T), reflecting the horizontal spread of the bleb, remained around 23–24 mm throughout follow‐up, while longitudinal extension (L), indicating the anteroposterior spread, stayed close to 17–18 mm after the first month. These findings suggest that early postoperative maturation is characterized mainly by vertical expansion, with the lateral and anteroposterior dimensions mainly established soon after surgery. Figure 1a,b depict representative postoperative courses to demonstrate different bleb developments (Figure 1).

**FIGURE 1:**
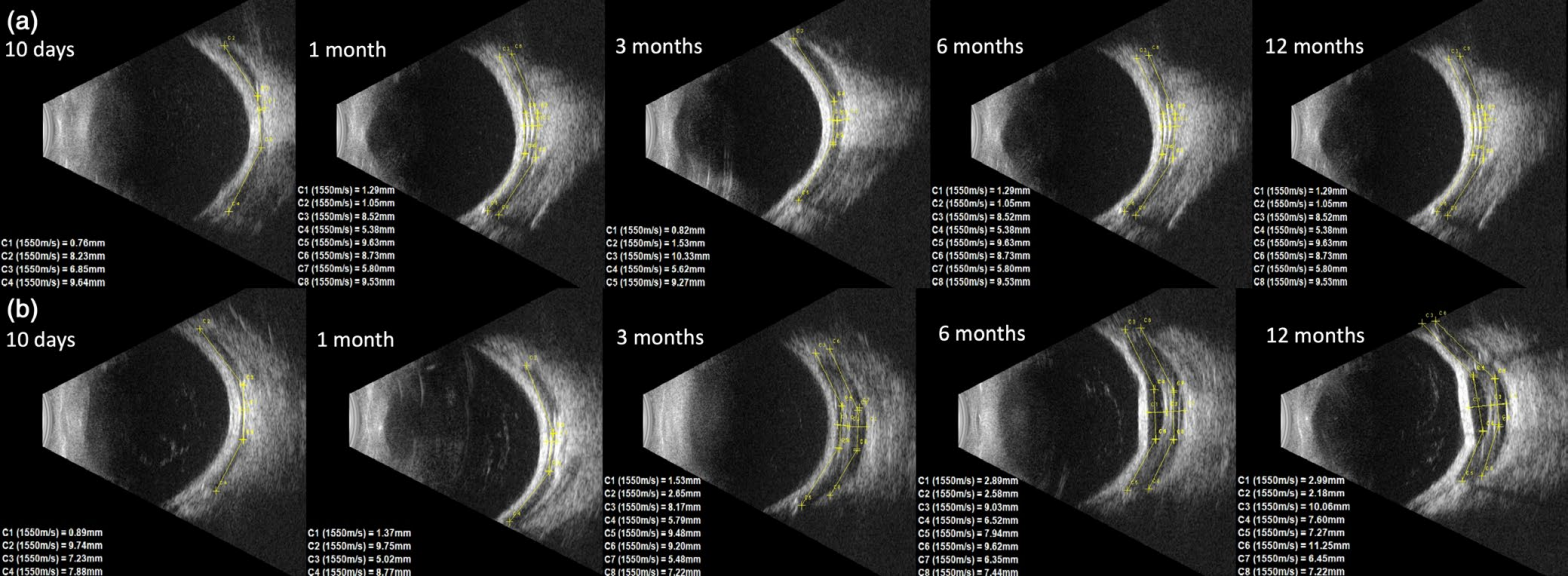
(a) Exemplary case 1: Postoperative course of the filtering bleb in a patient with secondary glaucoma after vitreoretinal surgery, in which a filtering bleb with a compartment beneath the plate is present after 14 days, reaching a plateau after 1 month and remaining stable thereafter. (b) Exemplary case 2: Postoperative course of the filtering bleb in a patient with secondary glaucoma after irradiation of an iris melanoma: Initially, the filtering bleb showed only one compartment, which developed into two compartments between the first and third months. From the sixth‐month onward, a plateau was reached, accompanied by stable intraocular pressure values around 12 mmHg without the need for additional pressure‐lowering medication.

Mean growth rates (mm/month) decreased steadily over time, indicating a slowing of growth with longer observation intervals. Between 14 days and 1 month, the mean rate was 1.57 ± 1.84 mm/month. Over 14 days to 3 months, it declined to 0.55 ± 0.61 mm/month and further to 0.27 ± 0.29 mm/month between 14 days and 6 months. By the 12‐month interval, growth was minimal, with a mean of 0.09 ± 0.17 mm/month (Table 2). These results indicate a progressive reduction in growth velocity over the course of the study period.

### 3.3 Correlation between bleb development and IOP

We performed mixed‐model analyses to account for correlations between bleb development and IOP. In the level models, absolute bleb height was significantly associated with higher IOP (*β* = 0.86, *p* = 0.043). Neither the postoperative time points (1–12 months) nor their interactions with bleb height reached statistical significance (all *p* > 0.10), except for a trend at 12 months, which approached statistical significance (height × time, *β* = −1.21, *p* = 0.058) (Figure 2). This suggests that absolute bleb height is a consistent predictor of IOP, largely independent of postoperative time (Table 3).

**FIGURE 2:**
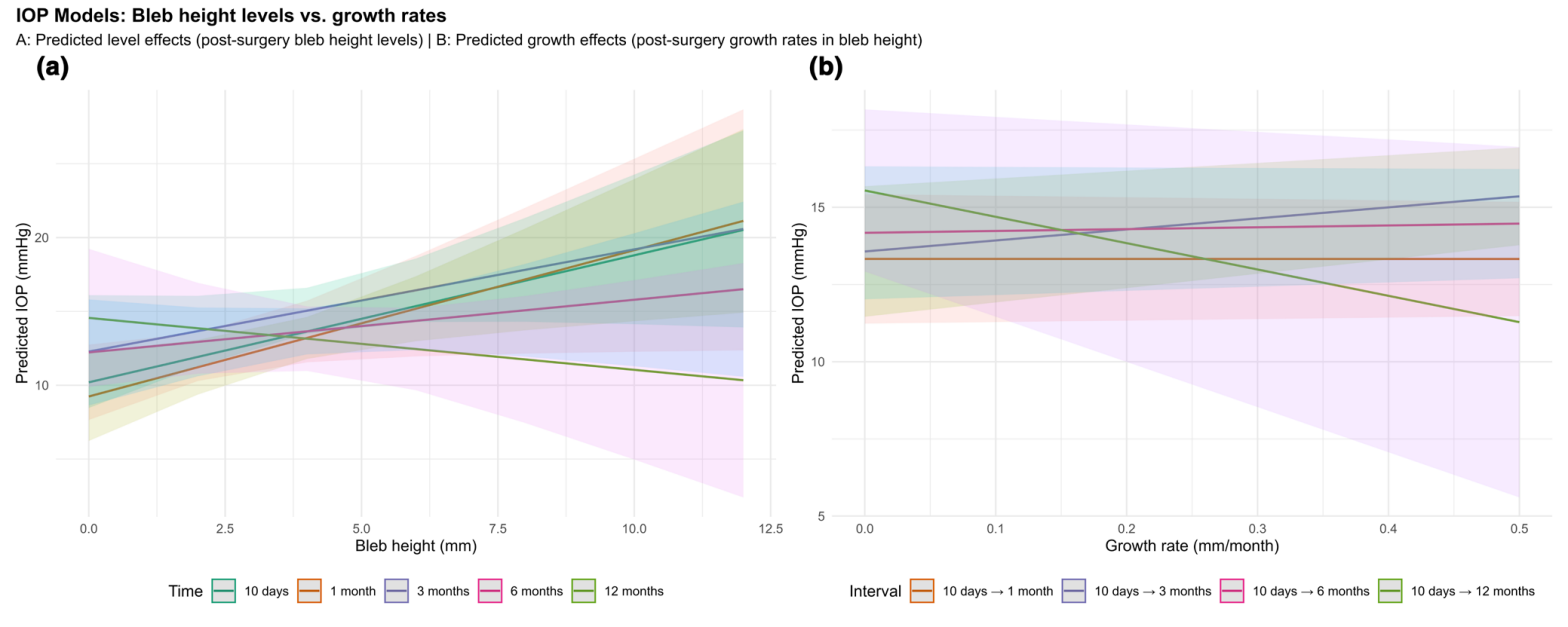
IOP models. Bleb height levels vs. growth rates: Left: Level effects (post‐surgery bleb height levels), right: Growth effects (post‐surgery growth rates of bleb height).

**TABLE 3:** Level models to check for the association between IOP and different parameters.

Characteristic	Beta	95% CI	*p*
Level models to check for association between IOP and different parameters			
Time			
14 days	—	—	
1 month	−0.96	−4.49, 2.57	0.592
3 months	2.08	−2.23, 6.39	0.341
6 months	2.02	−2.02, 6.07	0.324
12 months	4.36	−0.69, 9.42	0.090
Total bleb height	0.86	0.03, 1.69	0.043
Number of eye drops (0/1/2/3+)			
0	—	—	
1	0.00	−1.76, 1.76	0.998
2	0.66	−1.99, 3.30	0.625
3 or > 3	1.00	−1.34, 3.33	0.401
BCVA (logMAR)	−0.39	−1.87, 1.09	0.604
Sex			
Male	—	—	
Female	0.84	−0.81, 2.48	0.311
Age	−0.03	−0.07, 0.02	0.280
Time * Total bleb height			
1 month * Total bleb height	0.13	−0.93, 1.19	0.807
3 months * Total bleb height	−0.17	−1.31, 0.98	0.772
6 months * Total bleb height	−0.50	−1.58, 0.57	0.356
12 months * Total bleb height	−1.21	−2.47, 0.04	0.058
Growth model to check for the association between IOP and different parameters			
Interval			
14 days → 3 months	0.24	−2.22, 2.70	0.847
14 days → 6 months	0.84	−1.50, 3.19	0.476
14 days → 12 months	2.22	−0.51, 4.94	0.110
Growth rate	0.01	−0.78, 0.77	0.993
Baseline height	0.17	−0.43, 0.77	0.572
Number of eye drops (0/1/2/3+)			
0	—	—	
1	−0.66	−2.53, 1.22	0.490
2	0.43	−2.41, 3.27	0.764
3 or > 3	1.72	−1.13, 4.57	0.233
Visual acuity (logMAR)	0.38	−1.49, 2.24	0.689
Sex			
Male	—	—	
Female	−0.68	−2.70, 1.33	0.497
Age	−0.03	−0.08, 0.03	0.355
Growth Rate × Interval			
10 days → 3 months * Growth Rate	3.57	1.20, 5.95	0.003
10 days → 6 months * Growth Rate	0.60	−3.98, 5.17	0.797
10 days → 12 months * Growth Rate	−8.52	−21.22, 4.18	0.186

Level contrasts of slopes of bleb height development and growth rate during the postoperative course			
Contrast	Estimate	Std. error	*p* (DF)
Contrasts: Slopes of bleb height (per Time)			
14 days–1 month	−0.13	0.54	0.999 (14–5)
14 days–3 months	0.17	0.58	0.998 (15–6)
14 days–6 months	0.50	0.55	0.888 (14–9)
14 days–12 months	1.21	0.64	0.325 (15–8)
1 month–3 months	0.30	0.53	0.980 (14–7)
1 month–6 months	0.63	0.50	0.705 (14–3)
1 month–12 months	1.34	0.60	0.174 (15–5)
3 months–6 months	0.34	0.54	0.972 (14–4)
3 months–12 months	1.04	0.63	0.458 (15–2)
6 months–12 months	0.71	0.61	0.768 (15–3)
Contrasts: Slopes of growth rate (per interval)			
14 days → 1 month–10 days → 3 months	−3.57	1.21	0.020 (10–4)
14 days → 1 month–10 days → 6 months	−0.60	2.34	0.994 (11–0)
14 days → 1 month–10 days → 12 months	8.52	6.48	0.555 (10–2)
14 days → 3 months–10 days → 6 months	2.98	2.40	0.603 (95)
14 days → 3 months–10 days → 12 months	12.10	6.40	0.239 (98)
14 days → 6 months–10 days → 12 months	9.12	6.51	0.502 (92)

Abbreviations: CI, confidence interval; DF, degrees of freedom.

In the growth models, which accounted for postoperative growth rate, a more nuanced pattern emerged. No significant association between growth and IOP was observed within the first month (*β* = −0.004, *p* = 0.993). However, in the interval up to 3 months, accelerated growth was strongly associated with higher IOP (*β* = 3.57, *p* = 0.003). Later intervals (6 and 12 months) showed no consistent associations (all *p* > 0.18) (Table 3).

## 4 DISCUSSION

Sonography is a readily accessible imaging modality that enables detailed evaluation of bleb function following GDD surgery by visualizing the drainage area around the implant plate (Baig et al., 2015; Duan et al., 1997; Lloyd et al., 1993; Venkataraman & Mohan, 2022; Weber et al., 2024). To date, no studies have systematically examined the longitudinal development of GDD blebs using sonographic imaging. While ultrasound and MRI have been employed to visualize blebs after GDD surgery, existing research has been limited to cross‐sectional analyses, offering only single time‐point observations rather than tracking morphological changes over time (Anderson et al., 2017; Correia Barão et al., 2023; Iwasaki et al., 2017; Sano et al., 2015).

Our findings provide new insights into the typical course of bleb maturation, highlighting the usefulness of ongoing monitoring in the early postoperative period. At 2 weeks following surgery, nearly 40% of blebs presented as single‐layered structures—corresponding to the early modelling phase. However, this rapidly changed: by 1 month, the vast majority of eyes (87%) had developed double‐layered blebs, and the mean total bleb height had increased to almost 4 mm. Continued growth was seen over the 3 to 6 months postoperative period, when the mean total height reached about 4.2 mm, after which values remained stable through 12 months despite minor individual fluctuations. Transversal and longitudinal extensions were already broad at baseline—around 23 mm and 17 mm, respectively—and showed little further change, indicating that most lateral and anteroposterior expansion occurs early. This pattern suggests that most bleb formation and expansion occur within the first 3–6 months after GDD implantation. The stabilization observed at 12 months aligns with the hypothesis that once a bleb reaches its functional morphology, further expansion is unlikely and may not provide additional IOP‐lowering benefit. On a population level, this plateau phase appears stable and benign. Anderson et al. have published a longitudinal MRI imaging study in seven patients after Baerveldt implantation, showing bleb expansion between 1–2 weeks and 4–6 months postoperatively, confirming our findings and reinforcing the notion that the first months after surgery are critical for bleb formation and maturation (Anderson et al., 2017).

These longitudinal trends closely align with previously described bleb pathophysiology. Freedman reported that the plate of a tube shunt can stimulate the formation of a thin episcleral covering even before aqueous begins to drain. Once flow is established, this covering expands to form a functioning bleb, initially undergoing wall thickening due to postoperative inflammation and later thinning as degeneration processes take over (Freedman, 2021). Our early findings of predominantly single‐layered blebs may correspond to this inflammatory‐fibroproliferative phase. The subsequent development of double‐layered blebs with increased height may reflect the shift toward a more functional, fibro degenerative phase.

Histological studies of GDD blebs further support this trajectory. Molteno et al. showed that blebs with thinner walls and minimal fibroproliferation—characterized by superficial cell turnover and connective tissue breakdown in deeper layers—were associated with better filtration (Molteno et al., 2003, 2015). In contrast, dense fibroblastic activity and extracellular matrix accumulation, as described by Välimäki et al., were linked to impaired outflow and bleb failure (Välimäki & Uusitalo, 2014, 2015). Extrapolating from these findings, it is reasonable to infer that single‐layered blebs, particularly if persistent, may reflect an ongoing fibroproliferative response with activated fibroblasts, contributing to reduced filtration efficiency. Conversely, the earlier transition to double‐layered morphology may represent a more favourable remodelling pattern, enhancing bleb function and IOP control.

The mixed model revealed a consistent positive association between total bleb height and IOP across all follow‐up time points, indicating that patients with generally higher bleb elevations tended to exhibit higher IOP values. No significant time × height interactions were observed, suggesting that this relationship remained stable over the postoperative period. Thus, rather than reflecting transient fluctuations, the association between bleb morphology and pressure appears to represent a global structural trend that persists throughout follow‐up. While a negative association—larger bleb height corresponding to lower IOP—would be physiologically intuitive, our statistical analysis demonstrated a small but positive effect. This association indeed remained positive throughout all models (*β* = 0.86 mmHg per unit height), although the effect size was minimal and only marginally significant. This finding should therefore be interpreted with caution, as its clinical relevance is likely to be limited. Importantly, bleb height does not necessarily equate to functional filtration capacity. Particularly in the early postoperative period, greater bleb elevations may also reflect tissue reactions such as transudation, oedema or early fibrotic remodelling rather than improved aqueous outflow. Hence, the observed positive association may simply represent a subtle structural variation rather than a meaningful functional relationship.

Experimental studies have consistently demonstrated a relationship between bleb size and aqueous outflow capacity, with larger blebs generally associated with enhanced pressure‐lowering efficacy. As the bleb expands, it typically offers greater surface area for fluid absorption and diffusion, contributing to improved IOP control. However, this relationship is not linear indefinitely; rather, it appears to follow a diminishing‐return pattern, commonly referred to as a ‘ceiling effect’(Bouremel et al., 2019; Minckler et al., 1987). If a bleb still enlarges after that time, this might not be correlated with a better IOP‐lowering effect. This might be explained by the fact that beyond a certain size threshold, further increases in bleb volume may not lead to additional reductions in IOP. Instead, excessively large blebs can become counterproductive. Mechanical factors—such as stretching of the surrounding tissues, increased wall tension and collapse of adjacent compartments—may introduce internal resistance to aqueous flow (Minckler et al., 1987; Molteno et al., 2003; Wilcox & Kadri, 2007). Additionally, the overdistension of tissue planes may lead to microstructural changes within the bleb wall, including fibrosis or thickening, which can further impair fluid dynamics.

In addition, our results suggest that early postoperative growth dynamics are critical for treatment success. While the level model explores the static association between bleb morphology and IOP across time, the growth model explicitly quantifies the rate of morphological change. The growth model also highlights the critical importance of months 2–3 after surgery, during which accelerated bleb growth was associated with less favourable pressure control. For clinical practice, this may imply that patients should be monitored particularly closely during this period in order to allow timely therapeutic intervention.

Moreover, if rapid bleb growth is observed, an earlier tapering of topical corticosteroids could be considered as a theoretical approach to encourage a controlled degree of scarring. This might help limit excessive bleb enlargement while maintaining sufficient long‐term pressure control. However, corticosteroid tapering was not systematically performed in our cohort, and further studies would be needed to evaluate the clinical relevance of such an approach.

Clinically, this reinforces the value of longitudinal imaging: a rapidly enlarging bleb should not be assumed to equate with improved function. On the contrary, if IOP begins to rise despite ongoing bleb growth, it may indicate the onset of biomechanical resistance or early fibrosis. Identifying this trend early could allow timely intervention—whether through medical therapy, modulation of wound healing or surgical revision—to preserve long‐term pressure control. However, a decrease in bleb height or complexity in an individual eye—especially during or after the 6‐ to 12‐month window—may signal the onset of fibrotic remodelling or bleb failure. Such cases may benefit from early therapeutic intervention. While needling with 5‐fluorouracil (5‐FU) is not routinely performed by our group in the context of GDD surgery and was not applied in our cohort, it may represent a theoretical option to address subconjunctival fibrosis and maintain bleb morphology and function. Further studies are required to determine whether such interventions are appropriate or effective in this setting. Several studies have reported favourable IOP outcomes following needling procedures when bleb encapsulation is identified clinically (Eibschitz‐Tsimhoni et al., 2005; Quaranta et al., 2016). With the use of sonography, a progressive decrease in bleb height may serve as an early indicator of encapsulation—potentially before clinical signs become apparent. This could enable earlier and more targeted needling, possibly improving the efficacy of the intervention by addressing fibrosis before it becomes advanced or irreversible. Incorporating bleb imaging into routine postoperative monitoring may therefore support timelier and more individualized treatment strategies, optimizing surgical success and long‐term pressure control.

The ability to tailor postoperative interventions based on dynamic changes in bleb morphology represents a key strength of longitudinal sonographic evaluation. Unlike static assessments, which offer only a snapshot in time, serial imaging provides a nuanced understanding of each patient's healing trajectory. This individualized approach may be particularly beneficial in high‐risk or refractory glaucoma cases, where the margin for error is narrow.

This study has several limitations. Although our dataset includes longitudinal data, each eye was not imaged at uniform time intervals, and some time points had limited sample sizes. It also varied across time points because some patients were seen by their general ophthalmologist between scheduled visits and did not return to our clinic for intermediate assessments. Moreover, at the time of analysis, not all eyes had completed a full 12‐month follow‐up. Several patients who, in principle, would have reached the 12‐month visit were treated at distant centres and, given their stable IOP, did not return to our clinic. As a result, imaging at the 12‐month time point was not available for these cases, and they could not be included in the analysis. Loss to follow‐up may introduce bias, particularly if patients who did not return differ systematically from those who completed all visits. While mixed‐effects models tolerate missing data under the assumption that data are missing at random (MAR), this assumption cannot be fully verified and should be considered when interpreting the findings. Additionally, our observations were limited to a 12‐month follow‐up period. Extending the observation period would be valuable to obtain long‐term data on bleb behaviour. Thus far, blebs demonstrated progressive enlargement and appeared to reach a plateau in height; however, it remains unclear whether some may subsequently decrease in size over time, which could indicate fibrotic remodelling. Finally, while our cohort was limited to PGI implants, preliminary observations suggest similar trends may apply to other GDD types, such as Ahmed valves; this warrants further investigation.

## 5 CONCLUSION

In conclusion, longitudinal sonographic monitoring offers a valuable window into the biological evolution of GDD blebs. Most blebs develop their definitive morphology within the first 6 months, after which changes may indicate either stabilization or regression. Recognizing these patterns in real time can help guide proactive management, including antifibrotic therapy or staged stent removal, ultimately improving surgical outcomes and long‐term IOP control.

## FUNDING INFORMATION

CL received funding from the Ernst‐und‐Berte‐Grimmke foundation.

## CONFLICT OF INTEREST STATEMENT

CL, BA, LB, PS, MP, RL certify that they have no affiliations with or involvement in any organization or entity with any financial interest (such as honoraria; educational grants; participation in speakers' bureaus; membership, employment, consultancies, stock ownership or other equity interest; and expert testimony or patent‐licensing arrangements), or non‐financial interest (such as personal or professional relationships, affiliations, knowledge or beliefs) in the subject matter or materials discussed in this manuscript. KM has received honoraria or research grants from the following: Allergan/Abbvie (C, H, R) and Glaukos (C, H, R) but none of this is related to the current study. FGH has received honoraria or research grants from the following: Acucela (C, F), Allergan (F), Apellis (C, F), Bayer (C, F), Boehringer‐Ingelheim (C), Bioeq/Formycon (F, C), CenterVue (F), Ellex (F), Roche/Genentech (C, F), Geuder (C, F), Graybug (C), Gyroscope (C), Heidelberg Engineering (C, F), IvericBio (C, F), Kanghong (C, F), LinBioscience (C), NightStarX (F), Novartis (C, F), Optos (F), Oxurion (C), Pixium Vision (C, F), Oxurion (C), Stealth BioTherapeutics (C), Zeiss (F, C), but none of this is related to the current study.

## Data Availability

Deidentified individual participant data that support the findings of this study, along with the data dictionary and relevant analytic code, will be made available upon reasonable request to the corresponding author. Data will be provided for research purposes only and upon completion of a data‐sharing agreement. There are no publicly available repositories for these data due to institutional and ethical restrictions.
